# Understanding Idiopathic Spinal Cord Herniation – A Comprehensive Review of Imaging and Literature

**DOI:** 10.25259/JCIS-25-2019

**Published:** 2019-05-24

**Authors:** Pranav Sharma, Priti Soin, Mohamed Elbanan, Puneet Singh Kochar

**Affiliations:** 1 Department of Radiology, Yale New Haven Health Bridgeport Hospital, Bridgeport, Connecticut, USA; 2 Department of Pathology and Laboratory Medicine, Weill Cornell College of Medicine, New York, USA.

**Keywords:** Spinal cord herniation, Idiopathic spinal cord herniation, Dural defect, Cord kink

## Abstract

Idiopathic spinal cord herniation (ISCH) is displacement of spinal cord through a dural or arachnoidal defect. Most patients present with back pain or myelopathy, paresthesia, and sensory or motor weakness. Imaging findings include anterior displacement of the cord with possible kink, no filling defect on CT myelography, and no restricted diffusion/mass lesion on *magnetic resonance imaging*. Abrupt kink in the spinal cord or widened cerebrospinal fluid (CSF) space can be caused by a variety of reasons. The differential considerations include arachnoid web, intradural extramedullary epidermoid or arachnoid cyst, abscess or cystic schwannoma. We discuss the features, imaging, differentials, and treatment of ISCH as a rare cause of such kink in the cord. While reading such cases, a radiologist should include the location, segments involved, cord signal abnormality, visible defect, scalpel sign or C–sign, ventral cord kink, nuclear trail sign, the ventral CSF space preservation, or obliteration and the type.

## INTRODUCTION

Idiopathic spinal cord herniation (ISCH) is an uncommonly recognized cause of non-compressive myelopathy in which the spinal cord herniates through a small anterior or lateral dural defect.^[1]^ There is no associated history of trauma or surgery. Timely recognition is warranted to prevent permanent neurological deficits. Increased awareness of this clinical condition may result in a timely diagnosis and prompt treatment. In this review, we discuss clinical and an imaging characteristic of this rare pathology followed by a comprehensive review of literature.

## MATERIALS AND METHODS

Clinical and imaging data of three patients with ISCH were analyzed.

## CASES

### Case 1

**Figure 1:**
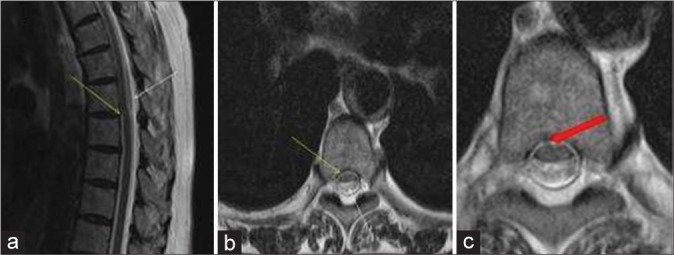
A 67-year-old female who presented with complains slowly progressing bilateral leg weakness with associated chronic low back pain. (a) Sagittal and (b) Axial T2-weighted image of the thoracic spine demonstrating anterior displacement of the spinal cord with an associated kink at T6 level (Yellow arrows) with CSF pulsation artifacts in the posterior subarachnoid space (White arrows). (c) High-resolution thin slice T2-weighted image demonstrates a subtle small focus of herniated cord through the dural defect (Red arrow). The findings favor spinal cord herniation instead of a posterior arachnoid cyst.

**Figure 2:**
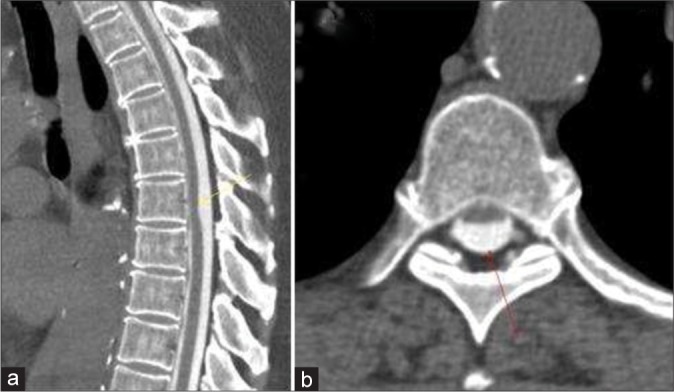
A 67-year-old female who presented with complains slowly progressing bilateral leg weakness with associated chronic low back pain. (a) Sagittal and (b) Axial CT Myelogram images of the thoracic spine demonstrating anterior cord kink at T6 (Yellow arrow) with complete opacification of CSF posterior to the anteriorly displaced spinal cord (Red arrow).

A 67-year-old female presented with complains slowly progressing bilateral leg weakness with associated chronic low back pain. There was no bowel or bladder involvement. On examination, motor and sensory exams were normal. Lab work was unremarkable. Magnetic resonance imaging (MRI) was performed which demonstrated anterior displacement of the spinal cord with an associated kink at T6 level with cerebrospinal fluid (CSF) pulsation artifacts in the posterior subarachnoid space [Figure 1]. High-resolution thin slice T2-weighted imaging demonstrated subtle small focus of herniated cord through the dural defect [Figure 1]. The findings favored spinal cord herniation instead of a posterior arachnoid cyst. To confirm the findings, a CT myelogram was performed which showed anterior cord kink at T6 with complete opacification of CSF posterior to the kinked and anteriorly displaced spinal cord confirming spinal cord herniation and excluding arachnoid cyst [Figure 2].

### Case 2

**Figure 3:**
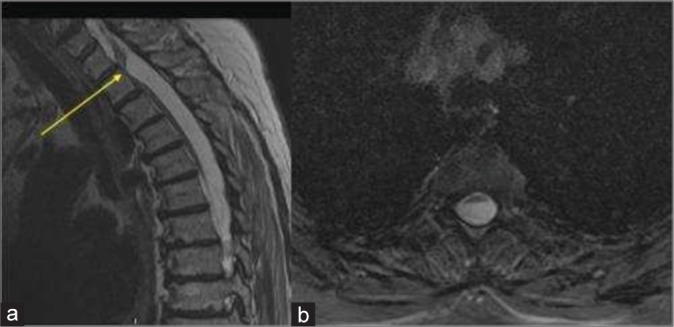
A 76-year-old female who presented with progressive onset difficulty in walking, leg weakness, and gait ataxia. (a) Sagittal T2-weighted and (b) Axial T2 gradient images of the thoracic spine demonstrating anterior cord kink at T2-3 level representing anterior spinal cord herniation (Yellow arrow).

A 76-year-old female presented with progressive onset difficulty in walking, leg weakness, and gait ataxia. On clinical exam, she had a sensory level T9 and T8-9 myelopathy was suspected. Lab parameters were within normal limits. There was no history of trauma. MRI thoracic spine demonstrated acute ventral kink in the thoracic spinal cord at T2-3 level [Figure 3].

### Case 3

**Figure 4:**
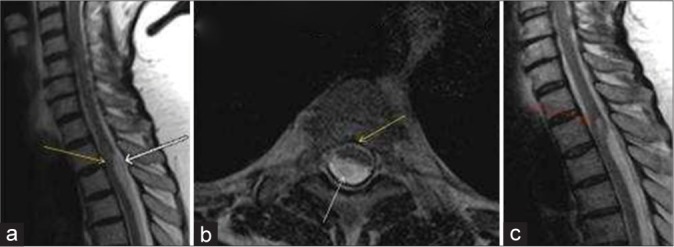
A 76-year-old female who presented with progressive onset difficulty in walking, leg weakness, and gait ataxia. (a) Sagittal and (b) Axial T2-weighted images of the thoracic spine demonstrating anterior cord displacement (“C” shaped kink) of the spinal cord with an associated kink at T4-5 level representing anterior spinal cord herniation (Yellow arrows). Posterior to the kink, CSF pulsation artifacts are seen in the posterior subarachnoid space (White arrows). (c) Sagittal T2-weighted image of the thoracic spine demonstrating a focal swelling with small intramedullary T2 hyperintensity is seen in the spinal cord just proximal to the kink representing a focal syrinx (Red arrow).

A 52-year-old female presented with progressive onset lower extremity weakness and gait abnormalities. Neurological exam was within normal limits. Lab parameters were unremarkable. She denied a history of trauma. MRI thoracic spine demonstrated acute ventral kink in the thoracic spinal cord at T4-5 level [Figure 4 a-c].

## DISCUSSION

### Etiology and pathogenesis

The etiopathogenesis of ISCH remains unclear; however, the focal dural defect has been blamed for the development of this rare clinical condition.^[2,3]^ Several mechanisms have been postulated to cause these dural defects. Remote history of trauma or trivial/occult injury may lead to dural tears.^[3]^ Herniated calcified disk may cause thinning, erosion, or rupture of the dura.^[3]^ In addition, it has been postulated that congenital duplication of dura can provide a potential space for the cord to herniate between the two layers of the dura.^[4]^ ISCH exclusively involves the thoracic spinal cord.^[3]^ This has been suggested due to a limited range of mobility of the thoracic spine and ventral spinal cord curve secondary to of physiologic kyphosis.^[5]^

## CLINICAL PRESENTATION

ISCH presents in 22–71 years of age^[6]^ with female predominance (M/F ratio of 3:2).^[7]^ Most common clinical presentation is Brown-Séquard syndrome, in greater than half of the reported cases.^[8]^ Other major early manifestations may include numbness and decreased temperature sensation in the legs, gait disturbances, pain, and incontinence.^[3,8]^

## IMAGING

MRI is the imaging modality of choice for diagnosing ISCH. Usually, there is a single dural defect; however, there have been reports of two defects.^[9]^

An abrupt anterior kink may be seen, called the “Scalpel Sign” [Figure 3 in our series] or there may be a gradual C- or S-shaped kink [Figure 4 in our series].^[1,10]^ The herniated part of the cord might appear as a small mass with a signal [Figure 1 in our series] or density similar to cord best seen on high-resolution MRI or CT myelography.^[1,11]^ Occasionally, abnormal cord signal abnormality may be seen representing syrinx formation [Figure 4 in our series] or myelomalacia of the spinal cord.^[12]^ Scalloping of the posterior vertebral body may also be noticed.^[12]^ The end plate irregularity and/or sclerosis or herniated disc calcification called “Nuclear trail Sign” can be seen sometimes on CT scan.^[13]^

The visualization of nerve roots traversing the dorsal subarachnoid space at the level of the cord deformity helps to differentiate SCH from other pathologies posterior to cord displacing it anteriorly such as a subarachnoid cyst or epidermal cyst which instead displaces the nerve roots peripherally.^[1]^ This is best appreciated on high-resolution T2-weighted images such as constructive interface in steady state or sampling perfection with application-optimized contrasts using different flip angle evolution.^[14,15]^ When the findings on MRI are equivocal, a CT myelography should be performed. It shows free migration of contrast through the dural defect at the level of the herniated segment or may show the widened dorsal subarachnoid space.^[12,16-18]^ The phase contrast pulse cine MRI demonstrates normal pulsatile CSF flow in subarachnoid space posterior to herniated cord in ISCH differentiating it from posteriorly positioned intradural arachnoid cyst.^[17]^

A classification system for ISCH has been reported based on the severity of herniation which helps in pre-operative planning and also in prognosticating patients.^[11,19]^

1. Type K: Kink toward the ventral region.
2. Type D: Cord disappears at the herniated site.
3. Type P: Protrusion of the cord in a way that the ventral subarachnoid space is effaced with very little posterior kink.
4. Type C: When the hiatus is central.
5. Type L: Hiatus is lateral.

The Type P has good post-operative recovery. Type C hiatus with bone defect have severe pre-operative symptoms and poor post-operative outcomes.^[11]^

## MANAGEMENT

The treatment of ISCH includes conservative management or surgery depending on the clinical presentation. Surgery is recommended with severe neurological symptoms or progressive worsening with the aim to reduce the herniated cord and prevent its recurrence.^[20,21]^ Surgery is laminectomy with intradural adhesiolysis and arachnoid band resection with verification of flow of CSF from cranial and dorsal ends of dural opening followed by closure of dural defect with fat or dural patch or widening of the dural defect.^[15]^ Surveillance is recommended for either form of treatment.^[22]^

## CONCLUSION

ISCH is a rare clinical entity with a displacement of the spinal cord through a dural or arachnoidal defect. Early recognition of this abnormality is necessary to prevent permanent neurological deficits. An important imaging finding is anterior displacement of the cord with a kink, without associated mass lesion.

## References

[1] Barrenechea IJ, Lesser JB, Gidekel AL, Turjanski L, Perin NI (2006). Diagnosis and treatment of spinal cord herniation: A combined experience. J Neurosurg Spine.

[2] Barbagallo GM, Marshman LA, Hardwidge C, Gullan RW (2002). Thoracic idiopathic spinal cord herniation at the vertebral body level: A subgroup with a poor prognosis? Case reports and review of the literature. J Neurosurg.

[3] Tekkök IH. (2000). Spontaneous spinal cord herniation: Case report and review of the literature. Neurosurgery.

[4] Sugimoto T, Kasai Y, Takegami K, Morimoto R, Maeda M, Uchida A (2005). A case of idiopathic spinal cord herniation with duplicated dura mater. J Spinal Disord Tech.

[5] Borges LF, Zervas NT, Lehrich JR (1995). Idiopathic spinal cord herniation: A treatable cause of the brown-sequard syndrome case report. Neurosurgery.

[6] Spissu A, Peltz MT, Matta G, Cannas A (2004). Traumatic transdural spinal cord herniation and the nuclear trail sign: Case report. Neurol Sci.

[7] Wortzman G, Tasker RR, Rewcastle NB, Richardson JC, Pearson FG (1974). Spontaneous incarcerated herniation of the spinal cord into a vertebral body: A unique cause of paraplegia. Case report. J Neurosurg.

[8] Massicotte EM, Montanera W, Ross Fleming JF, Tucker WS, Willinsky R, TerBrugge K (2002). Idiopathic spinal cord herniation: Report of eight cases and review of the literature. Spine (Phila Pa 1976).

[9] Aydin AL, Sasani M, Erhan B, Sasani H, Ozcan S, Ozer AF (2011). Idiopathic spinal cord herniation at two separate zones of the thoracic spine: The first reported case and literature review. Spine J.

[10] Reardon MA, Raghavan P, Carpenter-Bailey K, Mukherjee S, Smith JS, Matsumoto JA (2013). Dorsal thoracic arachnoid web and the “scalpel sign”: A distinct clinical-radiologic entity. AJNR Am J Neuroradiol.

[11] Imagama S, Matsuyama Y, Sakai Y, Nakamura H, Katayama Y, Ito Z (2009). Image classification of idiopathic spinal cord herniation based on symptom severity and surgical outcome: A multicenter study. J Neurosurg Spine.

[12] Najjar MW, Baeesa SS, Lingawi SS (2004). Idiopathic spinal cord herniation: A new theory of pathogenesis. Surg Neurol.

[13] Awwad EE, Martin DS, Smith KR (1992). The nuclear trial sign in thoracic herniated disks. AJNR Am J Neuroradiol.

[14] Grewal SS, Pirris SM, Vibhute PG, Gupta V (2015). Identification of arachnoid web with a relatively novel magnetic resonance imaging technique. Spine J.

[15] Schultz R, Steven A, Wessell A, Fischbein N, Sansur CA, Gandhi D (2017). Differentiation of idiopathic spinal cord herniation from dorsal arachnoid webs on MRI and CT myelography. J Neurosurg Spine.

[16] Darbar A, Krishnamurthy S, Holsapple JW, Hodge CJ (2006). Spine (Phila Pa 1976).

[17] Brugières P, Malapert D, Adle-Biassette H, Fuerxer F, Djindjian M, Gaston A (1999). Idiopathic spinal cord herniation: Value of MR phase-contrast imaging. AJNR Am J Neuroradiol.

[18] Adams RF, Anslow P (2001). The natural history of transdural herniation of the spinal cord: Case report. Neuroradiology.

[19] Haber MD, Nguyen DD, Li S (2014). Differentiation of idiopathic spinal cord herniation from CSF-isointense intraspinal extramedullary lesions displacing the cord. Radiographics.

[20] Groen RJ, Middel B, Meilof JF, de Vos-van de Biezenbos JB, Enting RH, Coppes MH (2009). Operative treatment of anterior thoracic spinal cord herniation: Three new cases and an individual patient data meta-analysis of 126 case reports. Neurosurgery.

[21] Sasani M, Ozer AF, Vural M, Sarioglu AC (2009). Idiopathic spinal cord herniation: Case report and review of the literature. J Spinal Cord Med.

[22] Summers JC, Balasubramani YV, Chan PC, Rosenfeld JV (2013). Idiopathic spinal cord herniation: Clinical review and report of three cases. Asian J Neurosurg.

